# Pharmacy professionals’ perceptions of their professional duties in the Ethiopian health care system: a mixed methods study

**DOI:** 10.1186/s40545-023-00656-8

**Published:** 2023-11-21

**Authors:** Ewunetie Mekashaw Bayked, Getachew Nigatu Taye, Segenet Zewdie, Teshager Aklilu, Mesfin Haile Kahissay, Husien Nurahmed Toleha

**Affiliations:** 1 Department of Pharmacy, College of Medicine and Health Sciences (CMHS), Wollo University, P.O. Box: 1145, Dessie, Ethiopia; 2 Department of Pharmacy, Dessie Comprehensive Specialized Hospital (DCSH), Dessie, Ethiopia; 3 Department of Pharmacy, College of Medicine and Health Science, Injibara University, Injibara, Ethiopia; 4 Department of Pharmaceutics and Social Pharmacy, School of Pharmacy, College of Health Sciences, Addis Ababa University, Addis Ababa, Ethiopia

**Keywords:** Perception, Pharmacy, Professional, Role, Health system

## Abstract

**Background:**

Pharmacy professionals are experts in therapeutic knowledge, experience, and skills that are used to ensure desired patient outcomes, utilizing the best available clinical evidence and interventions in collaboration with the health care team. They perceive themselves as a provider of technical, standardized, and individualized advice. The objective of this study was thus to assess the perception of pharmacy professionals towards their current professional roles in the health care system in Dessie, a city in the north-east Ethiopian region.

**Methods:**

A mixed-methods sequential explanatory study was used to assess the perception of pharmacy professionals towards their professional roles in Dessie city administration from December 15–30, 2019. The study participants were all pharmacy professionals working at health facilities in Dessie. Self-administered questionnaires were used to collect quantitative data, and face-to-face key informant interviews were used for qualitative data collection. Data were entered, processed, and analyzed using SPSS 25.0 statistical software, and thematic analysis was used for the qualitative exploration using QDA Miner Lite software (v2.0.7, free edition version).

**Result:**

The study had a 97.7% response rate. Of the 301 participants, 173 (57.5%) were male. Most of the participants had a positive perception, while 38 (12.6%) had a poor perception of their current professional roles. Lack of physical access, poor initiatives, poor communication skills, and a lack of administrative support for pharmaceutical care were statistically significant at a *p* value of 0.05 and a 95% confidence interval. From the qualitative data, two major themes emerged: perceived roles and determinants (perceived facilitators and barriers).

**Conclusion:**

Pharmacy professionals’ roles were found to be influenced by a lack of physical access, poor initiatives, poor communication skills, and poor administrator support. Pharmaceutical care requires everyone’s involvement in addressing these factors for successful performance and a better outcome and in considering perceived facilitators and barriers.

## Introduction

Pharmacy professionals (PPs) are drug information experts [1–3]. Their role is to meet all of the patient’s drug-related needs through direct patient care and in teamwork with other health workers [4, 5], including diagnosis of illnesses and drug prescribing [6]. Clinically, pharmacists provide patient care that optimizes medication therapy and promotes health, wellness, and disease prevention [5, 7]. The practice areas of PPs can be in the community, hospital, drug information service, industry, marketing, sales, regulation, academia, and drug distribution [8]. In hospitalized patients, the role of PPs emphasizes collaborative interaction [9, 10]. The role of pharmaceutical care services in saving lives and protecting public health is particularly relevant in resource-limited settings [11]. The clinical role of pharmacists, according to the "California School of Pharmacy and National Center for Health Service Research", is prescribing, providing, and applying direct patient care; documenting and reviewing drug use and consumption; and providing training and consulting [9]. However, the scope of the PPs' practice ranges from traditional services (producing and distributing drugs) to a contemporary role (pharmaceutical care) [12], especially in improving healthcare in society [13] as well as individual patient outcomes [5].

Pharmacy service is a central component of the health care delivery system and plays a role in improving treatment outcomes through the availability and rational use of quality, safe, and effective medicines [14]. Patient care involving PPs proved to be cost-effective because of treatment success, avoidance of adverse drug events, optimization of complex regimens, the design of adherence programs, and the recommendation of cost-effective therapies [11].

Despite the fact that the number of medicines has increased dramatically, with considerable challenges in controlling the quality and rational use of drugs, access to medicines of assured quality remains a major concern worldwide [15]. Due to the low involvement of PPs, irrational prescribing and the consumption of suboptimal or unsafe medicines have been widely documented in developing nations [16]. On the other hand, interdisciplinary teamwork will be strengthened only when PPs are active players [17]. However, their role has been jeopardized due to a mindset traditionally held by physicians [18]. Even the PPs themselves perceived that their current assignments were mainly logistical. This generates dissatisfaction, as some of their clinical activities are not recognized by other health care professionals. This dissatisfaction at work can be correlated with other negative effects such as absenteeism, work fatigue, poor quality of work, and reduced commitment to the profession. Any of these consequences may affect the way that other staff members and patients see the PPs and can therefore limit interactions with them [19].

Some PPs were less confident in pharmacists’ ability to improve therapeutic outcomes. This is due to the perception by many PPs that physicians discourage the involvement of PPs in many aspects of patient care [5]. There was a role conflict between nurses and PPs as a result of an issue with an unclear job description, which led to an ambiguous perception of PPs’ roles in the health care system [20]. The healthcare professionals considered the pharmacy job to be vital for society, and the majority of them had an excellent perception of PPs and their performance [21].

To give pharmaceutical care services effectively, understanding the PPs’ perception of their role is essential; that will facilitate the design of strategies to provide maximum care to the clients [22]. Involving trained clinical PPs in the healthcare team leads to clinically relevant and well-accepted optimization of medicine use in resource-limited settings [23]. Managing medicines safely, effectively, and efficiently is central to the delivery of high-quality care that is focused on the patient and gives value for money [5].

Evaluation of the PPs’ perceptions of their roles, as well as documentation and review of the patient care given by the PPs, are essential for the future plan of clinical pharmacy services [24]. The role of PPs as drug monitors and counselors on health promotion issues has great value in providing good health care to patients [25]. The pharmacy profession is currently advancing at a considerable pace, and new roles are being proposed and spread, not only by the profession itself but also by other health care professions and by national and international authorities and agencies [26].

Currently, in Ethiopia, the pharmacy profession has begun to shift its emphasis from product-oriented issues to patient-oriented informational and cognitive services. Since pharmacy service is an integral part of the health-care delivery system, there is a growing need for PPs to realize the recent changes in pharmacy practice worldwide. Although the perception of people regarding the traditional roles of PPs was weak, the Ethiopian Ministry of Health adopted in its guidelines that PPs should serve the patient directly [27].

Despite its drawbacks, there has recently been an increase in the number of clinical PPs working in the public sector in Ethiopia. Within its young and ambiguous stage of development, the pharmacy profession requires greater attention to achieve the objective of pharmaceutical care. As a result, knowing the perception of PPs towards their professional role will be essential to improving pharmacy practices in Ethiopia, particularly in Dessie city administration, since there is a growing need for PPs and pharmacy services due to the expansion of health institutions to cover the growing population in the city. Although various pieces of literature describe PPs’ perceptions of their professional roles, no strategies or systems have been developed to change such perceptions, which impede PPs’ activities with patient care. Therefore, the aim of this study was to explore the perception of PPs regarding their professional role according to the Ethiopian health care system in the Dessie city administration.

## Methods

### Study area and period

The study was conducted in Dessie city administration, North-East Ethiopia, from December 15–30, 2019. Formerly known as Lakomelza, Dessie was established in 1882 and is situated 401 km from Addis Ababa. It serves as the largest urban center in northeastern Ethiopia. The city's main religions are Islam, accounting for 58.62% of the population, and Orthodox Christianity, making up 39.92%. The dominant ethnic groups are Amhara (94.89%) and Tigre (3.79%). A significant majority of the population, 85.4%, resides within the city limits. Of this population, 51% is economically active, with an employment rate of 88% [28].

The city is home to several governmental and private health facilities, including hospitals, specialty centers, health centers, health posts, medium and specialized clinics, imaging centers, basic medical laboratory centers, pharmaceutical wholesalers, pharmacies, and drugstores. Due to the high population numbers in the city and around it, there is a growing need for health care practitioners in general and PPs in particular. In line with the huge number of pharmaceutical wholesalers and retail outlets that have been opened, the number of PPs in the city is also increasing. Dessie is thus a huge city with numerous private and governmental facilities that make it preferred for this study. Therefore, it was desirable to explore the perceptions of PPs toward their current professional roles.

### Study design

An explanatory-sequential study design was employed. A cross-sectional study was conducted in the first phase to assess PPs' perceptions of their current professional roles in the health care system and associated factors. This was followed by a descriptive-qualitative approach to deeply explore their perceptions of the roles. A descriptive qualitative research design is capable of answering questions such as what, who, where, and when. Thus, researchers pose these questions to individuals knowledgeable about the phenomenon, in this case, the key informants. Consequently, a descriptive qualitative study can enhance the depth of quantitative research [29].

We conducted the quantitative approach by December 15 and 16, 2019. The collected data had been entered into SPSS 25.0 during the data collection process, analyzed on December 17, 2019, and interpreted from December 17–19, 2019. Then, based on the result of the quantitative analysis, we conducted complementary descriptive qualitative research from 20 to 25, 2019 to deeply delve into the PPs perceptions of their roles in the Ethiopian healthcare system. For this approach, the data collection and analysis procedures were conducted simultaneously. Finally, we merged the results from both approaches and interpreted the findings from December 26–30, 2019 to provide comprehensive information about the issue of interest (PPs’ perceptions of their roles in the Ethiopian healthcare system).

### Participants and sample

All PPs, diploma and above, who had worked in Dessie for at least 6 months were included, while PPs who were guests and refused to participate in the study were excluded. There was no need to calculate the sample size because the number of participants was small. So, for the quantitative survey, all PPs who met the inclusion criteria and work in Dessie city administration health institutions (a total of 308) were interviewed. The questionnaire was distributed to all PPs in these health-care institutions who met the inclusion criteria. The questionnaire administered by the interviewer was filled out by the PPs in all health institutions.

Purposive sampling of pharmacy heads at the selected health institutions was used for the qualitative approach, and only 12 PPs were interviewed because of the saturation of data. The key informant interview method of data collection was used to collect qualitative data. Up until data saturation, which marks the point in data collection when no new issues or insights are found and data starts to repeat so that continuing data collection is unnecessary, the pharmacy heads from a few different health institutions were each individually interviewed based on the topic guide [30].

### Data collection techniques

Face-to-face interviews were used to collect quantitative data via a structured questionnaire. The questionnaire was adapted from previous studies and modified to address the objectives [23, 31–33]. Then, a pretest was conducted with 5% of the participants (*n* = 15) in Haik Administrative City. The questionnaire contains four parts: socioeconomic and sociodemographic variables; roles of PPs; perceptions of PPs towards their current roles in the health care system; and perceived barriers. The quantitative data were collected by five diploma PPs after adequate training regarding the data collection tools was given by the principal investigator for one day. Quantitative data collection took 2 weeks. The principal investigator coordinates and supervises quantitative data collection.

Qualitative data were collected by the principal investigator. The qualitative data were collected to provide additional information regarding the perceptions of PPs towards their professional roles. An interview guide was developed after extensively reviewing the literature. Arrangements for the time and place of interviews were made during the initial contact. Written consent was obtained from the participants prior to the interview. The interviews mainly focused on PPs’ perceptions of their professional roles. Probing questions were used where necessary, and the participants were given freedom to express their views at the start of the interview session. Each interview was conducted by the researcher at a time and place convenient for the PPs and lasted approximately 4–14 min. The interviews were carried out in isolated places that were situated near or around the participants' work settings. All the interviews were conducted in Amharic (the working language of the study area) on an audio device and transcribed verbatim into MS Word.

### Data processing and analysis

Quantitative data were entered, processed, and analyzed using SPSS 25.0 statistical software after cleaning the data. Different frequency tables, graphs, and descriptive summaries were used to describe the study variables. Logistic regression analysis was used to determine the significance of associations between dependent and independent variables.

The internal consistency of the questionnaire was tested using Cronbach's alpha coefficient. To identify the association of each independent variable with the outcome variable, we used a bivariate logistic regression model. In this analysis, variables with a *p*-value less than or equal to 0.2 and a 95% CI were incorporated into the multivariate model. During the multivariate analysis, variables with a *p*-value less than 0.05 and a 95% confidence interval were deemed significantly associated with satisfaction. We used Hosmer and Lemeshow's assumption of goodness of fit in the binary outcomes, confirming that the model was fit (*p* value = 0.847).

The factors were rated using a 5-point Likert scale tool, which was transformed into two categories for analysis: agree (strongly agree and agree) and disagree (not sure, disagree, and strongly disagree) and reported accordingly. Then, participants with good perception were defined as those who scored higher than the average of the various computed variables used to assess perception. Poor perception was defined as being below the average of various computed variables used to measure perception.

For the qualitative data, the authors inductively thematized the transcripts line by line. Early coding of the Amharic transcript occurred concurrently with data collection following multiple readings. All written transcripts were read several times to obtain an overall feeling for them. All sections of relevant original transcripts were translated into English to facilitate coding, and each transcript was coded line by line manually. Qualitative data were analyzed using the principles of thematic analysis. Data-driven coding was used, and codes were organized into themes. For each transcript, significant phrases or sentences that pertain directly to pharmacists' perceptions of their professional roles and their contributing factors were identified. Key informants’ profession, sex, and work experience were used to elucidate their verbatim portrayals. Key quotes were considered to be illustrative of the emerging themes. The data analysis in this research was coded, categorized, refined, and organized using QDA Miner Lite software (v2.0.7, free edition version). Then, the emerging patterns were discussed using inductive thematic analysis, which is a method for identifying, analyzing, and interpreting patterns of meaning across qualitative data [34, 35].

### Data quality control

The study used a conceptual framework to guide this exploration. Before actual data collection time, the tool was pretested on 5% of the samples (*n* = 15) at Hayik town health institutions, about 30 km from Dessie, for its correctness on different groups of the source population, and possible adjustments or modifications were made to the tool. For the reliability of the five-level Likert scale items, the mean Cronbach’s alpha was calculated to be 0.72, which is within the acceptable range. Before data collection, all of the data collectors were trained and had a similar concept of the questionnaire, and after data collection, the collected data were checked for completeness and accuracy of the information before analysis. More than one investigator was involved in this study: the principal investigator and the two supervisors. Both qualitative and quantitative methods were used to triangulate the findings. Member checking was done for two key informants by bringing the study findings back. To present the quantitative results, we followed the guidelines provided by "Strengthening the Reporting of Observational Studies in Epidemiology (STROBE)" [36]. The write-up of the report of the qualitative part was also guided by the “Standards for Reporting Qualitative Research (SRQR) Checklist” [37].

### Reflexivity

Reflexivity is the sensitivity to the researcher's own personal experiences, biases, and assumptions in shaping research output [38]. Accordingly, the data collector (GNT) realizes that the result of this research is the interaction between him and the research participants. He has a master's degree in SAP and years of experience in pharmacy practice as a head of pharmacy. His professional status provides strengths and limitations for this study. Thus, to keep the strengths and overcome the limitations, he was aware of insider bias and his conflicting roles. His experience and expertise could make him better equipped to conduct this research. However, he faced the challenge of being perceived as a powerful individual based on his position. Hence, to address such biases, he used open-ended questions and engaged in informal conversations with informants on other topics they themselves raised. He was also non-judgmental in the whole research process and applied the principle of bracketing. Thus, he was conscious of all stages of the research process.

### Ethical consideration

Ethical approval of the research proposal was obtained from the ethical review committee of Wollo University with reference number CMHS814/02/2012, and permission was obtained from the administrative body of each institution. Informed written consent was obtained from study participants to confirm their willingness to participate after explaining the objective of the study, and those who volunteered to participate were provided with the questionnaire. The respondents were notified that they have the right to refuse or terminate at any point of the interview. The information provided by each respondent was kept confidential throughout the research process, which was assured by excluding their names and respecting their right not to participate in the study. The same principle was applied to the qualitative data. Participants were interviewed in distraction-free locations, and the audio recordings were saved on a password-protected laptop, accessible only by the investigators. All related data were securely stored and maintained confidentially in this manner.

## Results

### Quantitative results

#### Sociodemographic characteristics of the participants

The study had a 97.7% response rate. Out of a total of 301 participants, 173 (57.5%) were male. The mean age of participants was 30.13 years, with an SD of 6.55 years, and most of the participants were from Amhara ethnic groups (263, or 87.4%). Regarding the marital status of the participants, 124 (41.2%), 161 (53.5%), and 16 (5.3%) were single, married, and divorced, respectively (Table 1).

**Table 1:** Sociodemographic characteristics of PPs (*n* = 301), Dessie city administration, 2019

Variables	Category	Frequency	Percent
Sex	Male	173	57.5
	Female	128	42.5
Age	20–30	196	65.1
	31–40	91	30.2
	41 and above	14	4.7
Religion	Orthodox	185	61.5
	Muslim	106	35.2
	Others*	10	3.3
Ethnicity	Amhara	263	87.4
	Tigre	19	6.3
	Oromo	8	2.7
	Others**	11	3.7
Marital status	Single	124	41.2
	Married	161	53.5
	Divorced	16	5.3
Educational level	Diploma	149	49.5
	Bachelor degree	141	46.8
	Master’s degree	11	3.7
Total work experience	0–5 years	143	47.5
	5.1–10 years	102	33.9
	> 10 years	56	18.6

Others* = Protestant; Catholic; others** = Gurage, Wolayta, Agew

#### Socioeconomic characteristics of the participants

**Fig. 1:**
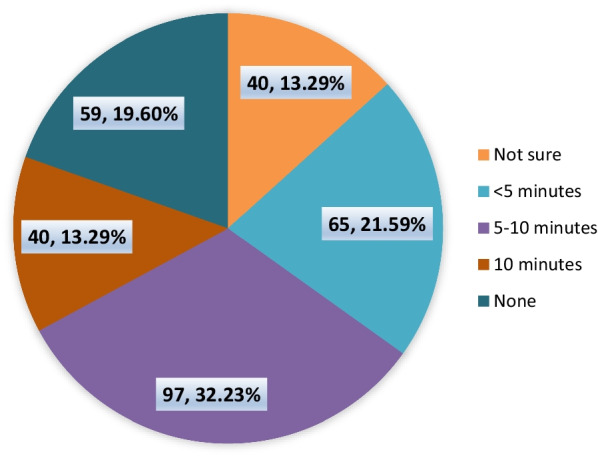
Average time spent by PPs (*n* = 301) with patients in Dessie city administration, north-east Ethiopia, 2019

From a total of 301 respondents, 49.5% were diploma holders, 46.8% were bachelor's degree holders, and 3.7% were master’s degree holders. Among the 301 study participants, 151 (50.2%) were government employees, and 150 (49.8%) worked in private institutions. The mean age of the participants was 6.8 years, with a standard deviation of 5.5 years. The mean monthly income of the participants was 5250.8 ± 2785.5 Birr. Ninety-seven (32.2%) of the PPs spent 5–10 min of their time with a single patient. The average time spent by PPs with a client is described in Fig. 1. In this category, none indicate that PPs have clearly measured their average time spent with clients.

**Fig. 2:**
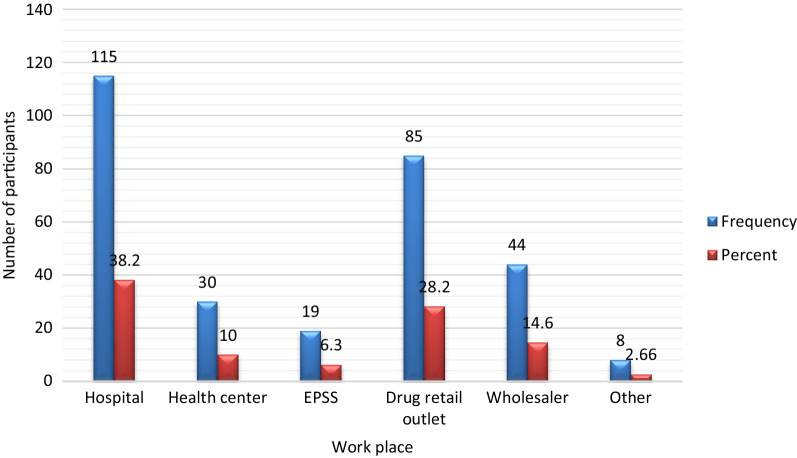
Workplace of the PPs (*n* = 301), Dessie city administration, north-east Ethiopia, 2019

As illustrated in Fig. 2, 114 (37.9%) of the pharmacists were working at the hospitals, followed by 85 (28.1%) at drug retail outlets and 44 (14.6%) at wholesalers.

**Fig. 3:**
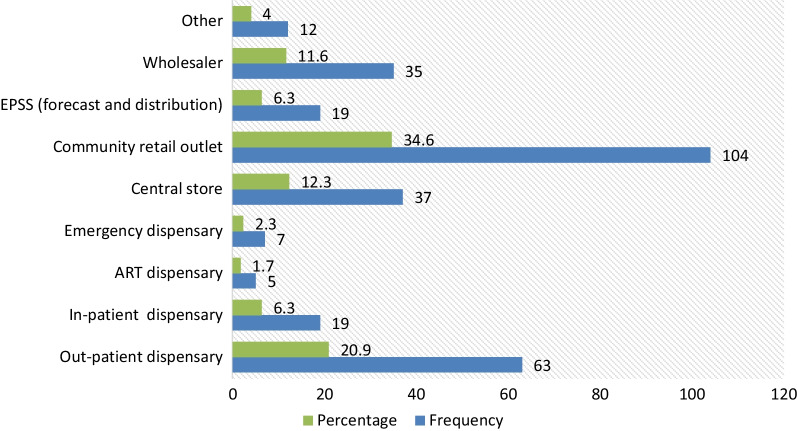
Working units of PPs (*n* = 301) in Dessie city administration, north-east Ethiopia, 2019

Regarding the current working units of the PPs in Dessie town, more than one-third (34.5%) were working at drug retail outlets, followed by those at outpatient department (OPD) pharmacies (20.9%) and stores (12.3%) (Fig. 3).

#### Roles of pharmacy professionals

By computing the ordinal responses of pharmacists' different activities in binary groups as poor perception (strongly disagree, disagree, and not sure) and good perception (agree and strongly agree) and adding all variables to take the average value, a single independent variable with binary outcomes is obtained. 203 (67.4%) of the participants perceived their roles as chemists, and 230 (76.4%) perceived themselves as clinicians or patient care providers (Table 2).

**Table 2:** Perception of PPs’ (*n* = 301) roles in Dessie city administration, north-east Ethiopia, 2019

Roles	Agree, No. (%)	Disagree, No. (%)
Chemist (compounder and manufacturer)	203 (67.4%)	98 (32.6%)
Clinician/care giver	230 (76.4%)	71 (23.6%)
Manager	229 (76.1%)	72 (23.9%)
Sales person	250 (83.1%)	51 (16.9%)
Communicator	256 (85.0%)	45 (15.0%)
Decision-maker	252 (83.7%)	49 (16.3%)
Teacher	236 (78.4%)	65 (21.6%)
Life-long learner	246 (81.7%)	55 (18.3%)
Researcher	229 (76.1%)	72 (23.9%)
Entrepreneur	234 (77.7%)	67 (22.3%)

#### Perceptions of pharmacy professionals towards their current roles

By dividing the ordinal responses of PPs' various activities into binary groups as poor perception (strongly disagree, disagree, and not sure) and good perception (agree and strongly agree) and adding all variables to take the average value, a single dependent variable with binary outcomes is produced, which could be useful for logistic regression analysis. One hundred seventy-eight (59.1%) of study participants strongly agreed that pharmaceutical care was in charge of providing medicine therapy. More than three-fourths (76.7%) of the participants agreed and strongly agreed that they perceived PPs’ roles as listening to patients' signs and symptoms in cases of minor illnesses. But the rest, 23.3%, disagreed about listening to patients' signs and symptoms in cases of minor illnesses. Dichotomized into binary outcomes, the dependent variable of this study was perception (1 = good perception, 0 = poor perception). Among 301 participants, 263 (87.4%) had a good perception and 38 (12.6%) had a poor perception of their current professional roles in the health care system in Dessie city administration health institutions. Each individual value is described in the following Table 3.

**Table 3:** PPs' (*n* = 301; after dichotomizing to binary outcomes) perceptions of their professional role in Dessie city administration in north-east Ethiopia, 2019

Statements	Good perception, No. (%)	Poor perception, No. (%)
Pharmaceutical care is the responsible provision of drug therapy	270 (89.7)	31 (10.3)
Pharmaceutical care is aimed at ensuring the safety, efficacy, economics, and rational use of medicines	271 (90.0)	30 (10.0)
Pharmaceutical care is just a medication counseling service	227 (75.4)	74 (24.6)
Listening to patients’ signs and symptoms in cases of minor illness	231 (76.7)	70 (233)
Dispensing proper medication to proper patients depending on their age, income level, and orientation	259 (86.0)	42 (14.0)
Asking every patient, a specific question about their medical history	233 (77.4)	68 (22.6)
Fill up prescription orders using the same trade names mentioned	182 (60.5)	119 (39.5)
Contacting doctors in cases of clarification or drug–drug interaction	230 (76.4)	71 (23.6)
Suggest the use of nonprescription medicine	211 (70.1)	90 (29.9)
Suggest the use of certain prescription medications to physicians	241 (80.1)	60 (19.9)
Identify and prevent prescription errors	261 (86.7)	40 (13.3)
Design and monitor pharmacotherapeutic regimens	254 (84.4)	47 (15.6)
Monitor the outcomes of pharmacotherapeutic regimens and plans	245 (81.4)	56 (18.6)
Involvement in the development of policies and guidelines for hospital regulations	229 (76.1)	72 (23.9)
Involvement in the compilation and updating of their hospital’s drug formulary	225 (74.8)	76 (25.2)
Only fill medication orders	184 (61.1)	117 (38.9)
Maintain the recordkeeping in the pharmacy	249 (82.7)	52 (17.3)
Willing to take personal responsibility for resolving drug-related problems	249 (82.7)	52 (17.3)
Cumulative perceptions	236 (78.43%)	65 (21.57%)

#### Perceptions of pharmacy professionals towards service-related factors

One hundred and eighty-four (61.1%) of the participants agreed that there is a shortage of physical access for pharmaceutical care provision, and the rest, 117 (38.9%), did not agree on this. Poor access to patients' clinical and laboratory data was agreed upon by 191 (63.5%) of the participants but not by 110 (36.5%). One hundred sixty-seven (55.5%) of the respondents agreed that there was effective communication skill among PPs, while the remaining 134 (44.5%) did not agree. One hundred fifty-one (50.2%) and 150 (498.8%) participants agreed and disagreed, respectively, that there was a lack of patient acceptance of pharmaceutical care provision. Lack of training among PPs was agreed upon by 187 (62.1%) and 114 (37.3%) of the participants (Table 4).

**Table 4:** Perception of PPs (*n* = 301), in line with pharmacy service-related factors, towards their professional roles in Dessie city administration, north-east Ethiopia, 2019

Variables	Agree, No. (%)	Disagree, No. (%)
Poor initiative for pharmaceutical care	162 (53.8)	139 (46.2)
Unclear scope of pharmaceutical care	131 (43.5)	170 (56.5)
Lack of confidence in pharmaceutical care	123 (40.9)	178 (59.1)
Poor cooperation with other health professionals	136 (45.2)	165 (54.8)
Lack of commitment to pharmaceutical care	150 (49.8)	151 (50.2)
Low compensation for pharmaceutical care provision	153 (50.8)	148 (49.2)
Shortage of pharmacy professionals	174 (57.8)	127 (42.2)
Poor expectations of pharmacy practice	164 (54.5)	137 (45.5)
The dispensary is far from the patient care area	175 (58.1)	126 (41.9)
Administrators do not support pharmaceutical care	182 (60.5)	119 (39.5)

#### Factors associated with the perception of pharmacy professionals towards their roles

The study found, after running binary logistic regression, that sex (*p*-value = 0.098), level of education (*p*-value = 0.17), experience of PPs (*p*-value = 0.062), lack of physical space for pharmaceutical care (*p*-value = 0.002), poor access to patient clinical and laboratory data (*p*-value = 0.061), lack of effective communication skills (*p*-value = 0.036), lack of training for PPs (*p*-value = 0.037), poor initiative for pharmaceutical care (*p*-value = 0.07), poor expectations of the pharmacy practice (*p*-value = 0.052), the dispensary's distance from the patient care area (*p*-value = 0.21), and lack of support from administrators (*p*-value = 0.056) were statistically significant at a *p*-value ≤ 0.2, and were candidates for the multivariate logistic regression (Table 5).

**Table 5:** Factors associated with the perception of PPs (*n* = 301) towards their professional roles in the Ethiopian health care system in Dessie city administration, north-east Ethiopia, 2019

Variables	Category	Perception		COR (95% CI)	AOR (95% CI)	*p*-Value
		Good, No. (%)	Poor, No. (%)			
Sex	Male	157 (52.15%)	16 (5.31%)	2.37 (1.02, 4.05)	1.77 (0.14, 1.98)	0.135
	Female	106 (35.21%)	22 (7.3%)	1	1	
Education level	Diploma	124 (41.16%)	25 (8.3%)	2.16 (1.18, 4.19)	1.64 (0.83, 3.32)	0.53
	Degree	130 (43.18%)	11 (3.65%)	2.21 (1.32, 3.90)	11.1 (3.49, 15.6)	0.61
	Master	9 (2.99%)	2 (0.66%)	1	1	
Experience	0–5 years	120 (39.86%)	23 (7.6%)	0.89 (0.39, 2.13)	0.58 (0.312, 1.115)	0.236
	5.-10years	91 (30.23%)	11 (3.65%)	5.72 (1.74, 18.6)	1.07 (0.457, 2.550)	0.056
	> 10 years	52 (17.27%)	4 (1.32%)	1	1	
Lack of physical access	Yes	95 (31.56%)	22 (7.3%)	0.411 (0.26, 0.82)	0.32 (0.049, 0.63)*	0.041
	No	173 (57.47%)	18 (5.98%)	1	1	
Poor access to clinical and laboratory data	Yes	90 (29.9%)	20 (6.64%)	0.48 (0.236, 0.90)	1.45 (0.630, 3.375)	0.27
	No	122 (40.53%)	12 (3.98%)	1	1	
Poor communication skill	Yes	141 (46.8%)	26 (8.63%)	0.93 (0.28, 5.02)	0.87 (0.18, 0.96)*	0.035
	No	168 (55.8%)	19 (6.31%)	1	1	
Lack of training	Yes	95 (31.56%)	19 (6.31%)	1.56 (1.285, 1.92)	0.36 (0.087, 1.495)	0.089
	No	146 (48.5%)	16 (5.31%)	1	1	
Poor initiatives	Yes	117 (38.87%)	22 (7.3%)	0.58 (0.293, 0.86)	0.25 (0.01, 0.97)*	0.012
	No	147 (48.83%)	17 (5.6%)	1	1	
Poor expectation of pharmacy practice	Yes	116 (38.5%)	21 (6.97%)	0.639 (0.32, 1.26)	1.59 (0.473, 5.394)	0.09
	No	157 (52.15%)	18 (5.98%)	1	1	
Dispensary far from patient care area	Yes	106 (35.2%)	20 (6.64%)	0.608 (0.37, 1.23)	1.48 (0.811, 2.732)	0.12
	No	168 (55.81%)	14 (4.65%)	1	1	
Administrator not support pharmaceutical care	Yes	95 (31.56%)	24 (7.97%)	0.68 (0.307, 1.23)	0.49 (0.16, 0.83)*	0.019
	No	157 (52.15%)	16 (5.31%)	1	1	

^*^Significant at a *p* value of less than 0.05

The following factors were not statistically significant: age (*p*-value = 0.6262), work experience (*p*-value = 0.35), marital status (*p* = 0.74), current working unit (*p*-value = 0.45), employer institution (*p*-value = 0.71), work place of the participant (*p*-value = 0.89), average monthly income (*p*-value = 0.44), lack of patient acceptance to pharmaceutical care (*p*-value = 0.74), unclear scope of pharmaceutical care (*p*-value = 0.85), lack of confidence in pharmaceutical care (*p*-value = 0.65), poor cooperation with other health care professionals (*p*-value = 0.77), lack of commitment to pharmaceutical care (*p*-value = 0.29), low compensation (*p*-value = 0.75), and a shortage of PPs (*p*-value = 0.73).

Hosmer and Lemeshow's assumption of model fitness was assessed, and the model fitness assumption was fulfilled (*p* = 0.847). Multiple logistic regressions were used as the final point of comparison in the analysis; enter the method for calculating variables and consider a *p* value of less than 0.05 to be statistically significant. Finally, those variables entered into multiple logistic regression were described in a regression table with their crude and adjusted odds ratios and 95% confidence intervals. Then, four variables were found to be statistically significant in multiple logistic regression: lack of physical access (*p*-value = 0.041; AOR = 0.32; 95% CI 0.049, 0.63); poor initiatives (*p*-value = 0.012; AOR = 0.25; 95% CI 0.01, 0.97); poor communication skill (*p*-value = 0.035; AOR = 0.87; 95% CI 0.18, 0.96); and the administrator does not support pharmaceutical care (*p*-value = 0.019; AOR = 0.49; 95% CI 0.16, 0.83).

One of the factors influencing PPs' perceptions of their current professional roles was a lack of physical space for pharmaceutical care. Those PPs who perceived a lack of space for pharmaceutical care had 0.32 (*p*-value = 0.041; AOR = 0.32; 95% CI 0.049, 0.63) times lower odds of good perception towards their current professional roles compared to those who perceived good physical space for pharmaceutical care, keeping all other variables constant.

PPs' effective communication skills were significantly related to their perceptions of their professional roles. Those PPs with poor communication skills had 0.87 times (*p*-value = 0.035; AOR = 0.87; 95% CI 0.18, 0.96) lower odds of having a positive perception of their professional roles compared to those with good communication skills, keeping all other variables constant.

The perception of PPs toward their professional roles in the health care system was significantly associated with the pharmaceutical care initiative. Those PPs with poor initiative for pharmaceutical care were 0.25 times (*p*-value = 0.012; AOR = 0.25; 95% CI 0.01, 0.97) less likely to have good perceptions towards their professional roles compared to those with poor initiative for pharmaceutical care, keeping all other variables constant.

Administrator support of pharmaceutical care was significantly associated with the perception of PPs towards their professional roles in the health care system. Those PPs who perceived a lack of administrator support were 0.49 times (*p*-value = 0.019; AOR = 0.49; 95% CI 0.16, 0.83) less likely to have a positive perception of their professional roles compared to those with poor initiative for pharmaceutical care, keeping all other variables constant.

### Qualitative findings

This study's qualitative findings yield two main themes: perceived PP roles and perceived determinants of their roles. The second theme, perceived determinants, was again categorized into two sub-themes: perceived facilitators and perceived barriers to the roles of PPs.

#### Sociodemographic descriptions of the participants

The mean age of the participants was 30 years. The key informants (*n* = 12) were all men. The educational status of participants was one diploma, ten bachelors, and one master. Regarding the employment status of the participants, seven were from governmental institutions and five were from private institutions. Table 6 shows that participants had a minimum of 2 years and a maximum of 12 years of experience.

**Table 6:** Sociodemographic descriptions of the key informants (n = 12), Dessie city, north-east Ethiopia, 2019

Code	Age	Education	Work area	Experience (year)
P1	29	Bachelor	Retail outlet	5
P2	27	Bachelor	Store	10
P3	30	Bachelor	OPD	5
P4	29	Bachelor	Retail outlet	4
P5	32	Bachelor	Retail outlet	8
P6	27	Master	Wholesale	6
P7	35	Bachelor	EPSS	12
P8	26	Diploma	OPD	2
P9	31	Bachelor	Retail outlet	5
P10	30	Bachelor	IPD	8
P11	27	Bachelor	OPD	5
P12	37	Bachelor	OPD	12

*EPSS* Ethiopian Pharmaceutical Supply Service, *IPD* Inpatient Department, *OPD* Outpatient Department

#### Perceived pharmacy professional roles

According to the study participants, various perceived professional roles were mentioned. These were coded into categories, such as counselor, researcher, leader, communicator, compounder, manufacturer, production manager, quality control, and technical manager. It was perceived that PPs are the core of the health care team, from dispensing to industry as well as research and development levels, especially in medicine production and manufacturing, including their rational utilization in patient care.

PPs play a variety of roles in the community, collaborating with other health care providers as researchers, teachers, compounding, counseling, dispensing, quality control, production management, leaders, and in industry. (P1).

In decreasing order of perceived frequency, the perceived roles were counselor, manager (drug supply, production, technical, and quality control), dispenser, teacher, chemist (as compounder and manufacturer), communicator, researcher, and leader. Participants most frequently described the counselor role, followed by the dispenser role.

We, the PPs, have different roles; one is counseling patients about their medications. (P1).

Consequentially, the finding revealed that some (*n* = 6) of the participants perceived their roles as dispensers and one-third of them as teachers.

Currently, patients come to PPs only to get the prescribed medicines; i.e., though PPs turn out to be involved starting with diagnostic procedures, patients do not come to PPs but rather their supporters. (P6).

When I see PPs in various locations, they do not do what is expected of them; rather, they dispense what is prescribed. (P12)

#### Perceived facilitators of pharmacy professional roles

Positive perceptions of PPs' roles and interactions with patients and other health care providers, including good opportunities and perceived encouraging impacts in the health service as a result of them (the PPs).

The era of clinical pharmacy practice that leads to good pharmaceutical care practices, being the source of drug information centers and increasing interaction with both clients and other health care providers, integrity and being honest for the profession, being updated using different learning opportunities, the scientific background that makes pharmacists drug experts, respected, and business opportunists were perceived as facilitators of the roles of PPs. Other perceived facilitators include the presence of regulatory bodies like the Ethiopian Food and Drug Administration (EFDA), the experience and professional alliance of PPs, adequate human power, supplies, and commodities.

According to key informants, being honest for the profession and respected by clients, the presence of a regulatory body like the EFDA, the work experience of the PPs, adequate human power and supply of commodities, pharmaceutical care practice, the interaction of the PPs with clients and other health care providers, being a business owner, professional updating, and the presence of a strong curriculum were perceived facilitators of the role of the PPs.

The image of the participants towards the pharmacy profession was positive in the past, serving the community, and negative currently, considered business-oriented, but most of the participants perceived a positive image towards their profession roles, which will facilitate professional practice.

According to the perceptions of the informants, the profession of pharmacy is the focal point of the health care delivery system and patient care.

Because they are drug specialists, many of the PPs are improving pharmacy services by performing what is expected in the community; they provide public services in the area of drug-related services that were needed; thus, the profession is the "backbone" of the health care system. (P1).

The ways to facilitate the roles of PPs for better future health care practice were self-respect, improving awareness and perceptions of pharmacy services in the community, having the employer organization fulfill materials for pharmacy services, having hard work and good performance of what is expected from PPs, having good interaction with other health care providers, having quality education for quality PP production, and having regular monitoring and evaluation by the regulatory body.

Because the pharmacy profession is respected in Ethiopia and around the world, it is important to respect oneself and have a positive image of oneself in order to serve the community in the future. (P1).

Quality education for PPs, regular monitoring and evaluation by regulatory bodies such as the EFDA, creating drug awareness among patients, and preparing various workshops for health care providers were all ways to improve pharmacists' perceptions for better future health care practice. (P7).

Most participants saw positive interactions between PPs and other health professionals, but few saw such interactions between PPs and patients.

We (the PPs) had positive interactions with nurses and physicians, especially in terms of communication on drug types and their dosages. (P8)

The majority of participants perceived a positive interrelationship between PPs and patients, but only a minority of participants perceived that interrelationship.

We (PPs) especially help patients by giving information on drug types, their doses, and how to take these drugs. (P8).

Being truthful about the profession and the recent launch of clinical pharmacy services aided pharmacy practice.

Serving patients anywhere with the best ethical discipline, knowledge, and skills is a good opportunity to practice the pharmacy profession. (P11)

The good will of the PPs, the current launch of the clinical pharmacy service, and the high number of PPs were opportunities to improve pharmacy practice. (P12)

The pharmacy service is essential to improving clinical care and patient outcomes, so its services can reduce negative impacts on patients. (P4)

#### Perceived barriers to pharmacy professional roles

The most commonly explored perceived barriers to the roles of PPs were lack of central focus, poor infrastructure, budget shortage, lack of administrative support, professional complexity, negligence of the PPs, and a high work burden due to a high patient load. Other perceived barriers were the lack of training for PPs, the PPs being business-centered, communication barriers, the absence of morning sessions, and the lack of departmentalization of different disciplines like dermatology, oncology, etc.

The lack of an effective central focus for PPs was one of the barriers to pharmacy practice.

Other health care providers and administrative staff, even employer organizations, did not understand the burdens of pharmacists. (P1)

Also, lack of administrative support and poor infrastructure were perceived barriers to the pharmacy practice.

The challenges for pharmacy practice were a shortage of materials and equipment, a lack of physical spaces, administrative issues like the low attitude of people in different positions within the pharmacy profession, and the absence of “unique departments" like dermatology and oncology. (P12).

Some of the study participants (*n* = 4) perceived barriers towards the profession of pharmacy because the practitioners in this field are only business-oriented, which negatively affects the implementation of pharmacy practice.

Currently, PPs are concerned with their business, while others are concerned with their professional service. When the profession is considered, the public benefits; when the business is considered, the public suffers. (P7).

Some other participants (*n* = 5) perceived that the interrelationship of PPs with patients differs from place to place, and these were barriers for the pharmacy profession. Some of the participants perceived a negative interrelationship between PPs and other health care providers.

We (the PPs) have poor interactions with patients in hospitals because it is a government facility with high patient flow and low PP satisfaction, but there is good interaction in private pharmacies because we work for business and want to bring them (the patients) as our regular customers. (P1).

The complexity of the profession and the lack of a clear job description were also barriers to the role of pharmacy practice.

PPs fear physicians because of the conflicting roles between them. (P1)

There is conflict with nurses since they prescribe for themselves. (P9)

One of the barriers to pharmacy practice was pharmacists' poor communication with patients and other health care providers.

Communication barriers were the practice's biggest challenge. (P9)

We (the PPs) have negative relationships with other health care providers. We isolated ourselves from other health care providers and had poor communication even after the start of clinical pharmacy services. (P5).

Negligence is one of the barriers that may hinder the practice of PPs’ roles.

There is some abandonment and contempt, as well as feelings of abandonment and contempt. (P5).

## Discussion

The role of PPs in the care of patients has evolved over time, with increased emphasis on collaborative care and patient interaction. The concept of "seven-star PP", detailing the roles each PP must perform, includes care-giver, decision-maker, communicator, manager, lifelong learner, teacher, and leader. In addition to the seven roles, the inclusion of them as researchers and entrepreneurs is quite significant [39]. To give pharmaceutical care services effectively, understanding the PPs’ perceptions of their role is essential; that will facilitate the design of strategies to provide maximum care to the clients. Evaluation of the PPs’ perceptions of their roles and reviews of the patient care given by them are essential for planning the future of pharmacy services.

Two hundred thirty-six (78.43%) of the PPs had a positive perception of their current professional roles in the health care system. The perception of PPs towards their professional roles in this study is higher compared to a study done in Indonesia, where only 20.1% of the PPs argued that they had a good perception of their roles [24]. This study revealed a lower perception of PPs towards their current professional roles compared to another study done in Malaysia, since all PPs except one had positive perceptions towards their roles [40]. In this study, 65 (21.57%) had poor perceptions of their current professional roles in the health care system. This may be due to the fact that some PPs were less confident about their ability to improve therapeutic outcomes. This is due to the perception by many PPs that physicians highly discourage their involvement in many aspects of patient care [5]. In addition, there were role conflicts between nurses and PPs as a result of an unclear job description and professional complex explored through a qualitative study, which led to an ambiguous perception of PPs' attitudes towards their role in the health care system [20]. Despite the above justifications, healthcare professionals considered the pharmacy job to be vital for society, which is why the majority of them had an excellent perception of pharmacists and their performance [21].

According to this study, 203 (67.4%) of the PPs perceived their roles as chemists, which is higher compared to a study done in the United Arab Emirates, where about half of the respondents (48.5%, *n* = 96) considered themselves chemists [41]. This was also supported by the qualitative exploration, which revealed that some of the participants perceived their roles as chemists (both compounding and manufacturing); the similarity in finding may be due to the fact that chemistry is also a core discipline of PPs related to medicine compounding and in industrial pharmacy as a manufacturer, as well as the international nature of the profession.

Two hundred thirty (76.4%) PPs saw themselves as pharmaceutical care providers, and 229 (76.1%) as managers. It was also perceived by most of the PPs that their roles were as clinicians/care givers and managers (as production manager, technical manager, drug supply, and quality control); similar to this study in technical tasks (care giver), but oppositely with regard to managerial tasks, a study done in Pakistan [22] said that the main focus of PPs was on services to patients rather than managerial tasks. A study done in Dubai showed that only 14.1% (*n* = 28) of the PPs perceived themselves as managers [41]. The discrepancy in perception of these roles may be due to the fact that the practical aspect of the pharmacy profession in our country is poorer than in others.

From the qualitative findings, most of the participants perceived their roles as dispensers and counselors. Similar studies also reported that PP roles were perceived as medication dispensing as per instructions and counseling [10, 22, 42]. However, a study in Indonesia showed that the roles of PPs were not only drug dispensing and consulting but also patient-care providers and health commodity suppliers [24]. In contrast to this, findings from a Canadian study show that PPs perceive themselves as guardians of medication and feel that their focus should be only on medications [43].

Our finding revealed that 250 (83.1%) of the PPs perceived themselves as salespeople and 234 (70.7%) as entrepreneurs. In line with this, another study showed that PPs perceived their roles as ranging from health-care professionals to businessmen [27]. According to a study conducted in Dubai, pharmacists are more focused on business than on patients. They virtually always dispense all types of drugs without requiring a prescription [41]. In fact, a very similar situation has been seen in our study area, which may lead to negative externalities unless effective regulation takes place.

According to our findings, 256 (85.0%) and 252 (83.7%) of the PPs perceived themselves as communicators and decision-makers, respectively. Two hundred thirty-six (78.4%), 246 (81.7%), and 229 (76.1%) of the study participants consider themselves to be teachers, lifelong learners, and researchers. Actually, PPs can learn from life experiences, both from their patients and their colleagues as health care providers [44, 45].

The study participants also perceived themselves as drug information sources, one of the roles of PPs. This may be due to the fact that the PPs are experts or specialists in drug information, so they can provide essential information both for clients and other health care providers [46].

Being honest with the pharmacy profession and having positive interactions with clients and other health care providers were mentioned as perceived facilitators for the successful implementation of PPs’ roles. A study conducted in Brazil found the same thing [47]. The pharmaceutical care practice was another facilitator, which was also shown by a study in England [48]. Adequate numbers of PPs who ease workload were one of the perceived facilitators, which was consistent with findings of studies conducted in Brazil [47], England [48], and British Columbia [49]. Educational opportunities were reported to be one of the perceived facilitators [11].

The lack of physical space for pharmaceutical care has a negative impact on PPs' perceptions of the health care system. PPs who perceive a lack of physical space for pharmaceutical care have a 0.32-times lower likelihood of being satisfied with their current professional roles. Similarly, according to a study conducted in Macao, lack of physical space and poor infrastructure are factors affecting the perception of PPs and pharmaceutical services [50].

A systematic review in Malaysia discovered that lack of funds was a barrier to pharmacy practice [51], which was also found in this study. Lack of understanding of PPs’ roles by other health care providers, administrative staff, and employer organizations was a barrier to their success. This was consistent with the findings of a study in Malaysia [51].

PPs' effective communication skills were significantly related to their perception of their professional roles. PPs with poor communication skills had 0.87 times lower odds of having a good perception of their professional roles than those with good communication skills. This was supported by the qualitative findings that lack of effective communication with clients and other health care providers is a major challenge for pharmacists. PPs face communication, integration, and interaction barriers in their roles. Another Ethiopian study also found that poor cooperation and conflicts of interest between pharmacists and other health care providers are perceived barriers to PP roles [1]. Studies conducted in China [52], Kuwait [18], Macao [50], and Brazil [47] also revealed that the lack of effective communication skills of PPs with patients and other health care providers was one of the perceived barriers to their roles. A qualitative study identified three key barriers to PPs providing clinical services: poor integration, issues around behaviors and cultures, and weak communication [53]. Conversely, effective communication is essential for client counseling and education, as well as cooperation with other health care providers [44, 45].

The initiative for pharmaceutical care was significantly associated with PPs' roles in the health care system. PPs with poor initiative for pharmaceutical care are 0.25 times less likely to have a good perception of their professional roles. Studies conducted in Thailand [54] and Brazil [47] also found that a lack of initiatives was one of the perceived barriers to PPs and pharmaceutical care.

Administrative support of pharmaceutical care was associated with a positive perception of PPs in the health care system. PPs who perceived a lack of administrative support were 0.49 times less likely to have a positive perception of their professional roles. Similarly, in key informant interviews, lack of administrative support and lack of central focus are challenges for PPs. This may be due to the fact that healthcare administrators have a misperception of the services provided by PPs; they are not providing the PPs with positive reinforcement, which leads to dissatisfaction and negatively affects their professional roles [55].

## Limitations

The study did not consider the perceptions of other health professionals. It did not also consider the perceptions of patients and the community regarding the role of PPs.

## Practical implications

Pharmaceutical care is essential for better patient health outcomes, and medication therapy is the most commonly used form of treatment. Pharmaceutical care is the responsible provision of drug therapy to improve a patient's quality of life. Its use has grown as the population has aged, chronic disease has increased, new infectious diseases have emerged, and the range of effective medications has increased. The PPs’ jobs are to interact with the health care team, interview and assess patients, make specific therapeutic recommendations, monitor patient responses to drug therapy, and provide medicine information [15]. Therefore, this is because good communication is essential for PPs to collect, synthesize, and interpret relevant information [56]. The study identified the perceived roles of PPs and factors affecting them that will benefit employers as well as the community.

## Direction to future research

Exploring the perceptions of other health care professionals and patients about PPs' roles and responsibilities is of paramount importance.

## Conclusion

About three-fourths of the pharmacy professionals involved in the study had a positive perception of their current professional roles in the healthcare system of the country. The main roles described in the qualitative findings were counselor, clinician, manager, dispenser, compounding teacher, researcher, quality control distributor, salesperson, and communicator. Lack of physical access, a lack of initiative, a lack of communication skills, and a lack of administrative support were factors that had a statistically significant association with pharmacy professionals' perceptions towards their professional roles. In decreasing order of perceived frequency, the perceived roles were counselor, manager (drug supply, production, technical, and quality control), dispenser, teacher, chemist (as compounder and manufacturer), communicator, researcher, and leader. Participants most frequently mentioned the counselor role, followed by the dispenser role. Being honest for the profession, being respected by clients, the presence of a regulatory body like the EFDA, the work experience of the PPs, adequate human power and supply of commodities, pharmaceutical care practice, interaction of the PPs with clients and other health care providers, being a business owner, professional updating, and the presence of a strong curriculum were perceived facilitators of the role of the PPs. The most commonly explored perceived barriers to the roles of PPs were lack of central focus, poor infrastructure, budget shortage, lack of administrative support, professional complexity, negligence of the PPs, and a high work burden due to a high patient load.

## Data Availability

The data that support the findings of this study are available within the article.
